# Transbronchial Needle Aspiration in the Staging of Bronchogenic Carcinoma

**DOI:** 10.1155/DTE.3.61

**Published:** 1996

**Authors:** Emil Benov, Krassimir Michev, Dimitar Kostadinov, Vesselin Vlasov

**Affiliations:** University Hospital of Pulmonary Diseases Bronchology Department Dimitar Nestorov str. 19 Sofia 1431 Bulgaria

## Abstract

To evaluate the usefulness of transbronchial needle aspiration biopsy (TBNA) for the diagnosis of mediastinal involvement, we have prospectively examined 316 patients with morphologically verified bronchogenic carcinoma. The percentage of positive aspirations (149 of 316) from the three basic lymph node groups in the mediastinum was not significantly different. Tumor cells were aspirated from the mediastinum in 75 of 112 patients with radiologically positive findings and in patients with 74 of 204 radiologically negative findings. Mediastinal involvement was verified even in 61 of 196 patients with a normal endoscopic picture. Metastases were proved in 14 of 39 patients with peripheral versus 135 of 277 patients with central carcinoma. Tumor cells were aspirated in 47 of 76 patients with undifferentiated small cell carcinoma, 92 of 227 patients with squamous cell carcinoma, and 10 of 13 patients with adenocarcinoma. Our results suggest that TBNA being a highly diagnostic and less invasive method, will prove its clinical importance.


*Diagnostic and Therapeutic Endoscopy*, Vol. 3, pp. 61–65
Reprints available directly from the publisher
Photocopying permitted by license only

© 1996 OPA (Overseas Publishers Association)
Amsterdam B.V. Published in The Netherlands
by Harwood Academic Publishers GmbH
Printed in Singapore

### Transbronchial Needle Aspiration in the Staging of Bronchogenic Carcinoma

EMIL BENOV, KRASSIMIR MICHEV*, DIMITAR KOSTADINOV and VESSELIN VLASOV

*University Hospital of Pulmonary Diseases, Bronchology Department, Dimitar Nestorov str. 19 Sofia 1431, Bulgaria*

*(Received 1 August 1995; In final form 25 March 1996)*

**To evaluate the usefulness of transbronchial needle aspiration biopsy (TBNA) for the diagnosis of mediastinal involvement, we have prospectively examined 316 patients with morphologically verified bronchogenic carcinoma. The percentage of positive aspirations (149 of 316) from the three basic lymph node groups in the mediastinum was not significantly different. Tumor cells were aspirated from the mediastinum in 75 of 112 patients with radiologically positive findings and in patients with 74 of 204 radiologically negative findings. Mediastinal involvement was verified even in 61 of 196 patients with a normal endoscopic picture. Metastases were proved in 14 of 39 patients with peripheral versus 135 of 277 patients with central carcinoma. Tumor cells were aspirated in 47 of 76 patients with undifferentiated small cell carcinoma, 92 of 227 patients with squamous cell carcinoma, and 10 of 13 patients with adenocarcinoma. Our results suggest that TBNA being a highly diagnostic and less invasive method, will prove its clinical importance.**

*Keywords:* Bronchoscopy, lung cancer, mediastinal metastases, transbronchial needle aspiration

### INTRODUCTION

Many factors influence the prognosis of patients with pulmonary carcinoma. One of the most important factors is the existence of metastases in the mediastinal lymph nodes. Based on the 5-year survival rate, a number of authors consider the involvement of the mediastinum, even when the resection is technically possible, to be a contraindication for surgical treatment [1–9]. Therefore, the evaluation of regional lymph nodes is an important part of the preoperative preparation of patients.

Lately, some authors mainly American, suggested the use of transbronchial needle aspiration (TBNA), introduced by Schieppati in 1958 [10], for morphological verification of mediastinal adenopathy. This method was first introduced using the rigid bronchoscope [11–18]. The development of the flexible technique and flexible needles by Wang and Terry in 1983 [17] and Oho *et al.* in 1979 [19] was a prerequisite for the use of the fiberoptic bronchoscope for TBNA [18,20,21]. Another development in this biopsy technique is the introduction of needles to obtain material for histological analysis both by rigid [22] and flexible [23] bronchoscopes.

*Corresponding author.

The aim of this study was to evaluate the usefulness of TBNA for the diagnosis of mediastinal involvement in patients with bronchogenic carcinoma, using two different techniques.

### MATERIALS AND METHODS

A prospective examination of 316 patients (58 women and 258 men) with primary pulmonary carcinoma was carried out. The diagnosis was morphologically verified in all cases. The lesion was localized in the left lung in 50 patients and in the right lung in 266 patients.

TBNA was performed as previously described [18–22] in 151 of the patients using a flexible needle type IIB (Mill Rose). TBNA was performed prior to the other biopsy procedures to avoid false-positive results [18,24]. We followed the endobronchial markers described by Wang *et al.* in 1984 [25] for increasing efficacy and decreasing the risk of damage of vital mediastinal structures. In the remaining 165 patients, TBNA was performed using a rigid bronchoscope (Storz) with needles N 10436 and N 10438, especially designed for this purpose.

All procedures were performed under local anesthesia with lidocaine after premedication with atropine when there were no contraindications.

### RESULTS

Of the 316 patients examined TBNA showed tumor cells in the regional lymph nodes in 149 (47.15%). The hilar lymph nodes have been examined in a relatively low number of patients, because their involvement in the neoplastic process does not have the same significance as lymph nodes localized in the mediastinum. In these patients the percentage of positive results was high: 34 of 37 or 91.89%.

The results of TBNA were not significantly related to the localization of the lymph nodes examined. The percentages of positive aspirations from the three basic groups of lymph nodes in the mediastinum were similar (Table I).

TABLE I Correlation of TBNA Findings with Localization of Mediastinal Lymph nodes

| Localization of Lymph Nodes | No. Punctures | Results: Positive *n* | Positive % | Negative *n* | Negative % |
|---|---|---|---|---|---|
| Subcarinal | 290 | 104 | 35.86 | 186 | 64.14 |
| Right paratracheal | 171 | 66 | 38.60 | 105 | 61.40 |
| Left paratracheal | 31 | 8 | 25.81 | 23 | 74.19 |
| Total | 492 | 178 | 36.18 | 314 | 63.82 |

Using TBNA, tumor cells from the mediastinum were more frequently aspirated in the patients with undifferentiated small cell carcinoma (47 of 76, 61.84%) and adenocarcinoma (10 of 13, 76.92%) and rarely when the primary tumor was of the squamous cell histologic type (92 of 227, 40.53%) (Table II). From 39 aspirations performed in patients with peripheral pulmonary carcinoma 14 (35.9%) had metastases in the mediastinum, versus 135 of 277 (48.74%) of the patients with central lesions (Table III).

Two other criteria, the radiologic view of the mediastinum and endoscopic findings, were compared with the diagnostic yield of TBNA. Using this method, in 66.96%, of the patients with radiologically positive findings and in 36.27% of the patients with radiologically negative findings, metastases in the mediastinal lymph nodes were identified. In 73.33% of the patients with endoscopic views suspicious for compression from mediastinal lymphadenopathy, tumor cells were obtained. We were successful using TBNA in verifying involvement of the mediastinal lymph nodes by the neoplastic process even in patients with a normal endoscopic picture (31.12%) (Tables IV and V).

TABLE II Results of TBNA Compared with the Histology of the Tumor

| Histology | No. Patients | Results: Positive *n* | Positive % | Negative *n* | Negative % |
|---|---|---|---|---|---|
| Squamous cell | 227 | 92 | 40.53 | 135 | 59.47 |
| Adenocarcinoma | 13 | 10 | 76.92 | 3 | 23.08 |
| Undifferentiated Small cell | 76 | 47 | 61.84 | 29 | 38.16 |
| Total | 316 | 149 | 47.15 | 167 | 52.85 |

TABLE III Efficacy of TBNA Related to Localization of Primary Tumor

| Localization | No. Patients | Results | | | |
|---|---|---|---|---|---|
| | | Positive | | Negative | |
| | | *n* | % | *n* | % |
| Central | 277 | 135 | 48.74 | 142 | 51.26 |
| Peripheral | 39 | 14 | 35.90 | 25 | 64.10 |
| Total | 316 | 149 | 47.15 | 167 | 52.85 |

For evaluating the usefulness of the new flexible technique in performing the procedure, the results obtained were compared with those from conventional rigid bronchoscopy. The efficacy is similar for both (Table VI) and the flexible technique is more convenient both for the patient and for the operator.

No significant complications from the procedure were observed. Minimal bleeding that always resolved spontaneously occurred at some of the puncture sites.

### DISCUSSION

Mediastinoscopy has quickly replaced other biopsy methods [26–28] after its introduction in 1959 [29]. Its high diagnostic value was proved in the 1960s [30]. Three groups of lymph nodes, anterior mediastinum, subaortic, and back carinal, however, remain inaccessible for cervical mediastinoscopy [31]. More important, the lymphatic drainage from the left lower lobe crosses the subcarinal lymph nodes and turns right, which is not the same for the left upper lobe [32]. Thus, left parasternal mediastinotomy has to be considered for the assessment of regional adenopathy in malignant lesions involving the left upper lobe of the lung [33].

TABLE IV Relationship of TBNA to Radiological Findings for Mediastinum

| Mediastinum | No. Patients | Results | | | |
|---|---|---|---|---|---|
| | | Positive | | Negative | |
| | | *n* | % | *n* | % |
| Normal | 204 | 74 | 36.27 | 130 | 63.73 |
| Abnormal | 112 | 75 | 66.96 | 37 | 33.04 |
| Total | 316 | 149 | 47.15 | 167 | 52.85 |

TABLE V Relationship of TBNA to Bronchoscopic Appearance of Airway

| Finding | Patients | Results | | | |
|---|---|---|---|---|---|
| | | Positive | | Negative | |
| | | *n* | % | *n* | % |
| Normal | 196 | 61 | 31.12 | 135 | 68.88 |
| Abnormal | 120 | 88 | 73.33 | 32 | 26.27 |
| Total | 316 | 149 | 47.15 | 167 | 52.85 |

TABLE VI Efficacy of TBNA Related to the Technique

| Technique | No. Patients | Results | | | |
|---|---|---|---|---|---|
| | | Positive | | Negative | |
| | | *n* | % | *n* | % |
| Rigid | 165 | 89 | 53.94 | 76 | 46.06 |
| Flexible | 151 | 60 | 39.74 | 91 | 60.26 |
| Total | 316 | 149 | 47.15 | 167 | 52.85 |

Mediastinoscopy has proved to be a reliable technique for staging and defining the resectability of pulmonary carcinomas. However, being a surgical intervention, it can be accompanied by serious complications [33,34] although recently these have been significantly reduced due to improved surgical and anesthesiologic techniques. The complication were the reason for introduction of alternative noninvasive techniques. While conventional roent-genography and tomography are not very useful [35–38] computed tomography (CT) is extremely sensitive [39–45]. Magnetic resonance imaging has almost the same sensitivity [46–49]. However, all imaging methods cannot distinguish the type of process, i.e., malignant or inflammatory, and this makes their predicative value quite low. So an additional morphologic verification is required. For this reason some authors [39–44] do not favor mediastinoscopy in patients with negative CT scans. They consider its application obligatory when enlarged lymph nodes are found in the attempt to minimize false-positive results with CT.

The role of TBNA in the preoperative evaluation of the mediastinum in the patients with pulmonary carcinoma is currently under evaluation. Its high sensitivity and specifity permit a reliable assessment of the spread of the neoplastic process in the mediastinum [18,20,21].

We can estimate the role and the significance of preoperative TBNA to assess the influence of mediastinal lymph nodes involvement on the operability of patients with primary lung cancer. Although the frequency of the positive results from the hilar lymph nodes is higher, far more important is the examination of level N2. The involvement of the hilar lymph nodes can result from direct invasion of a central lesion, and more important they remain in the resected piece of the lung. The extirpation of the mediastinal lymph nodes is much more difficult and largely defines the operability of the patient.

The more frequent finding of mediastinal metastases from nondifferentiated carcinoma and adenocarcinoma compared with squamous cell histologic type carcinoma could be explained by the higher metastatic potential. The manipulation, however, is beneficial regardless of the primary tumor type, because in 40.53% of patients with squamous cell histologic type carcinoma tumor cells have been found in the material obtained.

The relatively high percentage of positive results in patients with peripheral lung cancer (35.90%) deserves special attention. Regardless of the factors influencing these results (cytologic type, degree of expansion), this seems sufficient evidence to suggest the use of TBNA for staging even in patients with peripheral lung cancer. Thus, many unjustified thoracotomies in patients considered operable using other methods of staging can be avoided.

The radiologic and endoscopic findings as an indication for TBNA are of limited usefulness in our experience. This conclusion is based on the fact that of the patients with mediastinal metastases proved by TBNA 31.12% have a normal endoscopic picture and 36.27% show no radiographic evidence of mediastinal involvement. Therefore, the lack of radiologic and bronchoscopic signs for mediastinal lesions cannot be considered a contraindication for TBNA.

The results obtained by the conventional rigid technique, although somewhat better, are not statistically significantly different from those using the flexible technique. The easier and deeper penetration is an advantage of the rigid technique, which, however, cannot compensate for its being more traumatic and uncomfortable for the patient. The flexible technique, except for its other advantages, allows also a more punctual directing of the tip of the needle. For these reasons we prefer the use of flexible TBNA, performed under conditions that allow intubation with the rigid technique for the management of eventual bleeding.

In summary, TBNA is a highly effective method for the morphologic verification of mediastinal metastases in primary lung cancer or malignant lesions in the mediastinum. TBNA is indicated in patients with negative radiologic or endoscopic findings for evaluation of mediastinal involvement too. Of course, the introduction of needles allowing histologic specimens to be obtained will extend the possibilities of the method. We believe that TBNA, being a less invasive method with high diagnostic accuracy, will prove its importance in clinical practice.

### *References*

[1] Ashrah, M. H., Milson, P. L., Waleshy, R. K. (1980). Selection by mediastinoscopy and long-term survival in bronchial carcinoma, *Ann Thorac Surg*, **30**, 208.

[2] Beargh, N. P., Scherstien, T. (1965). Bronchogenic carcinoma, *Acta Chir Scand Suppl*, 347.

[3] De Larue, S. J. (1967). A review of some important problems concerning lung cancer, *Can Med Assoc J*, **96**, 8.

[4] Fosburg, R. G., O'Sullivan, M. J., Ah-Tye, P., *et al.* (1974). Positive mediastinoscopy. An ominous finding, *Ann Thorac Surg*, **18**, 346.

[5] Gunn, S. W., Ross, C. A. (1960). Effect of exploratory thoracotomy on the life expectation of patients with non-resectable carcinoma of the lung, *Can Med Assoc J*, **83**, 1029.

[6] Inberg, M. V., Klossner, Y., Linua, M. Y., *et al.* (1972). The role of mediastinoscopy in the treatment of lung carcinoma, *Scand J Thorac Cardiovasc Surg*, **6**, 293.

[7] Sarin, C. L., Nohl-Oser, H. C. (1969). Mediastinoscopy: a clinical evaluation of 400 consecutive cases, *Thorax*, **24**, 585.

[8] Shields, T.W., Jee, J., Conn, J.H., *et al.* (1975). Relationship of cell type and lymph node metastasis to survival after resection of bronchial carcinoma, *Ann Thorac Surg*, **20**, 501.

[9] Vincent, R. G., Takita, H., Lane, W. W., *et al.* (1976). Surgical therapy of lung cancer, *J Thorac Cardiovasc Surg*, **71**, 581.

[10] Schieppati, E. (1958). Mediastinal lymph node puncture through the tracheal carina, *Surg Gynaecol Obstet*, **110**, 243.

[11] Bridgman, A. H., Duffield, G. D., Takaro, T. (1968). An appraisal of newer diagnostic methods for intrathoracic lesions, *Dis Chest*, **53**, 321–327.

[12] Fox, R. T., Lees, W. M., Shields, T. W. (1965). Transcarinal bronchoscopic needle biopsy, *Ann Thorac Surg*, **1**, 92–96.

[13] Lemer, J., Malberger, E., Konig-Nativ, R. (1982). Transbronchial fine needle aspiration, *Thorax*, **37**, 270–274.

[14] Simecek, C. (1966). Cytological investigation of intrathoracic lymph nodes in carcinoma of the lung, *Thorax*, **21**, 369–371.

[15] Versteegh, R. M., Swierenda, J. (1963). Bronchoscopic evaluation of the operability of pulmonary carcinoma, *Acta Otolaringol (Stockh)*, **56**, 603–611.

[16] Wang, K. P., Terry, P., Marsh, B. (1978). Bronchoscopic needle aspiration biopsy of paratracheal tumors, *Am Rev Respir Dis,* **118,** 17–21.
[17] Wang, K. P., Marsh, B. R., Summer, W. R., *et al.* (1981). Transbronchial needle aspiration for diagnosis of lunf cancer, *Chest,* **80,** 48–50.
[18] Wang, K. P., Terry, P. B. (1983). Transbronchial needle aspiration in the diagnosis of bronchogenic carcinoma, *Am Rev Respir Dis,* **127,** 344–347.
[19] Oho, K., Kato, H., Ogawa, I., *et al.* (1979). A new needle for transfiberoptic bronchoscopic use, *Chest,* **76,** 492.
[20] Haponic, E. F., Wang, K. P. (1983). New methods for diagnosis and staging of mediastinal desease. In: Aisner J., ed: Lung Cancer. New York: Churchill Livingstone, Inc.
[21] Wang, K. P., Brower, R., Haponic, E. F., *et al.* (1983). Flexible transbronchial needle aspiration for staging of bronchogenic carcinoma, *Chest,* **84,** 571–576.
[22] Wang, K. P., Britt, E. J., Haponic, E. F., *et al.* (1985). Rigid transbronchial needle aspiration biopsy for hystological specimens, *Ann Otol Rhinol Laryngol,* **94,** 382–385.
[23] Wang, K. P. (1985). Flexible transbronchial needle aspiration biopsy for histologic specimens, *Chest,* **88,** 860–863.
[24] Shenk, D. A., Bryan, C. L., Brower, J. H., Myers, D. L. (1987). Transbronchial needle aspiration in the diagnosis of bronchogenic carcinoma, *Chest,* **92,** 83–85.
[25] Wang, K. P., Gupta, P. K., Haponic, E. F., *et al.* (1984). Flexible transbronchial needle aspiration: technical consideration, *Ann Otol Rhinol Laryngol,* **93,** 233–236.
[26] Daniels, A. C. (1949). A method of biopsy useful in diagnosing certain intrathoracic diseases, *Dis Chest,* **16,** 360.
[27] Harken, D. E., Black, H., Clausse, R., *et al.* (1954). A simple cervico-mediastinal exploration for tissue diagnosis of intrathoracic desease, *N Engl J Med,* **251,** 1041.
[28] Radner, S. (1955). Suprasternal node biopsy in lymphspreading intrathoracic disease, *Acta Med Scand,* **152,** 413.
[29] Carlens, E. (1959). Mediastinoscopy: a method for inspection and tissue biopsy in the superior mediastinum, *Dis Chest,* **36,** 343.
[30] Carlens, E., Hambreus, G. (1967). Mediastinoscopy, *Scand J Respir Dis,* **48,** 1.
[31] Pearson, F. G. (1968). An evaluation of mediastinoscopy in the management of presumably operable bronchial carcinoma, *J Thorac Cardiovasc Surg,* **55,** 617.
[32] Nohl-Oser, H. C. (1972). An investigation of the anatomy of the lymphatic drainage of the lung, *Ann R Coll Surg Engl,* **51,** 157.
[33] Foster, E. D., Munro, D. D., Dobel, A. R. C. (1972). Mediastinoscopy: a revew of anatomical relationships and complications (collective review), *Ann Thorac Surg,* **13,** 273.
[34] Ashbaugh, D. G. (1970). Mediastinoscopy, *Arch Surg,* **100,** 568.
[35] Libshitz, H. J., McKenna, R. J., Haynie, T. R., *et al.,* (1984). Mediastinal evaluation in lung cancer, *Radiology,* **151,** 295–299.
[36] McKenna, R. Jr, Libshitz, H. J., Mountain, C. E., *et al.* (1985). Roentgenographic evaluation of mediastinal nodes for preoperative assessment in lung cancer, *Chest,* **88,** 206–210.
[37] Moak, G. D., Cockerill, E. M , Fazber, M. O., *et al.* (1982). Computed tomography versus radiology in the evaluation of mediastinal adenopathy, *Chest,* **82,** 69–75.
[38] Faling, M. R., Pope, T. L. Jr. (1987). The variable nature of the mediastinal contour lines: CT/chest radiography correlation, *J Comput Tomogr,* **11,** 254–260.
[39] Baron, R. L., Levitt, R. G., Sagel, S. S., *et al.* (1982). Computed tomography in the preoperative evaluation of bronchogenic carcinoma, *Radiology,* **145,** 727.
[40] Breyer, R. H., Karstaedt, N., Mills, S. A., *et al.* (1984). Computed tomography for evaluation of mediastinal lymph nodes in lung cancer: correlation with surgical staging, *Ann Thorac Surg,* **38,** 215.
[41] Faling, L. J., Pugatch, R. D., Jun-Legg, J., *et al.* (1981). Computed tomographic scanning of the mediastinum in staging of bronchogenic carcinoma, *Am Rev Respir Dis,* **124,** 690.
[42] Goldstraw, P., Kuzzer, M., Edwards, D. (1983). Preoperative staging of computed tomography versus mediastiniscopy, *Thorax,* **38,** 10.
[43] Osborne, D. R., Korobkin, M., Havin, C. E., *et al.* (1982). Comparison of plain radiography, conventional tomography and computed tomography in detecting intrathoracic lymph node metastasis from lung carcinoma, *Radiology,* **142,** 157.
[44] Rea, H. H., Shevland, J. E., House, A. J. S. (1981). Accuracy of computed tomographic scanning in assessment of the mediastinum in bronchial carcinoma, *J Thorac Cardiovasc Surg,* **81,** 825.
[45] Underwood, G. H., Hooper, R. G., Axelbaum, S. P., *et al.* (1979). Computed tomographic scanning of the thorax in the staging of bronchogenic carcinoma, *N Engl J,* Med **300,** 777.
[46] Cassamasima, F., Villary, N., Fargonoli, R., *et al.* (1988). Magnetic resonance imaging and high-resolution computed tomography in tumors of the lung and mediastinum, *Radiother Oncol,* **11,** 21–29.
[47] Poon, P. J., Bronskill, M. J., Hankelman, R. M., *et al.* (1987). Mediastinal lymphnode metastases from bronchogenic carcinoma: detection with MR imaging and CT, *Radiology,* **162,** 651–656.
[48] Webb, W. R., Yensen, B. G., Sollito, R., deGreer, G., *et al.* (1985). Bronchogenic carcinoma: staging with MR compared with staging with CT and surgery, *Radiology,* **156,** 117–124.
[49] Webb, W. R. (1988). MR imaging in the evaluation and staging of lung cancer, *Semin Ultrasound CT MR,* **9,** 53–66.

